# CLIP-Llama: A New Approach for Scene Text Recognition with a Pre-Trained Vision-Language Model and a Pre-Trained Language Model

**DOI:** 10.3390/s24227371

**Published:** 2024-11-19

**Authors:** Xiaoqing Zhao, Miaomiao Xu, Wushour Silamu, Yanbing Li

**Affiliations:** College of Computer Science and Technology, Xinjiang University, No. 777 Huarui Street, Urumqi 830017, China

**Keywords:** scene text recognition, vision-language model, pre-trained language model

## Abstract

This study focuses on Scene Text Recognition (STR), which plays a crucial role in various applications of artificial intelligence such as image retrieval, office automation, and intelligent transportation systems. Currently, pre-trained vision-language models have become the foundation for various downstream tasks. CLIP exhibits robustness in recognizing both regular (horizontal) and irregular (rotated, curved, blurred, or occluded) text in natural images. As research in scene text recognition requires substantial linguistic knowledge, we introduce the pre-trained vision-language model CLIP and the pre-trained language model Llama. Our approach builds upon CLIP’s image and text encoders, featuring two encoder–decoder branches: one visual branch and one cross-modal branch. The visual branch provides initial predictions based on image features, while the cross-modal branch refines these predictions by addressing the differences between image features and textual semantics. We incorporate the large language model Llama2-7B in the cross-modal branch to assist in correcting erroneous predictions generated by the decoder. To fully leverage the potential of both branches, we employ a dual prediction and refinement decoding scheme during inference, resulting in improved accuracy. Experimental results demonstrate that CLIP-Llama achieves state-of-the-art performance on 11 STR benchmark tests, showcasing its robust capabilities. We firmly believe that CLIP-Llama lays a solid and straightforward foundation for future research in scene text recognition based on vision-language models.

## 1. Introduction

In the field of artificial intelligence, reading text from natural scene images, known as Scene Text Recognition (STR), is an essential capability for building intelligent systems. STR automatically recognizes text within natural images, such as street signs, billboards, and product labels. STR applications span numerous fields, including industrial automation, image-based geolocation, document analysis, human–computer interaction, image retrieval, and intelligent transportation systems. However, STR faces significant challenges due to the diversity and complexity of natural scene text, such as complex backgrounds, varied fonts, flexible arrangements, and occlusions. While traditional Optical Character Recognition (OCR) techniques have made notable progress in handling standard printed text, they often fall short when dealing with irregular text in natural scenes, such as rotated, curved, blurred, or obscured text.

In recent years, notable advancements in computer vision and natural language processing have propelled the rapid development of STR. Leveraging advanced deep learning architectures, large-scale annotated datasets, and algorithmic innovations, state-of-the-art STR methods continuously push the boundaries of accuracy and robustness, driving further applications and advancements in this dynamic field. STR remains a highly demanding task due to the inherent challenges posed by complex backgrounds, diverse fonts, flexible layouts, and unexpected occlusions in scene text, especially in challenging scenarios. Previous approaches have addressed these challenges by incorporating related tasks into the text recognition framework, leveraging additional information to improve recognition performance. Recently, a new trend has emerged in introducing language knowledge into the text recognition process. SRN [1] designed a global semantic reasoning module to model global semantic context, while ABINet [2] proposed a bidirectional prediction network to learn bidirectional feature representations as a language model. Both SRN and ABINet adopt standalone language models to capture rich linguistic prior knowledge. This approach, which combines visual models with language models [3], has shown improved performance in STR tasks. Some multitask and multimodal machine learning algorithms also provide valuable references for this task, such as methods DACT-GAN [4], MTLHand [5], and DSTFS [6]. In recent years, the development of Vision-Language Models (VLM) [7,8] and Large Language Models (LLM) [9,10] has provided new technological support for STR. These large models exhibit significant generalization abilities across various multimodal tasks, especially in understanding fine-grained visual content. For example, vision-language models such as CLIP [11], trained on nearly 400 million real image–text pairs, employ a multitask learning approach to simultaneously optimize image and text representations, aligning them more closely within the feature space. This establishes a tight embedding relationship between image and text, allowing for effective recognition and understanding of text information in natural scenes. Additionally, research has demonstrated that incorporating text semantics into the text recognition process can effectively enhance the model’s comprehension and predictive accuracy. Notably, including language models helps address uncertainty in character prediction, particularly when facing occluded or blurred text. Considering the substantial benefits of large language and vision models, we decided to conduct further research using pre-trained vision and language models. This study proposes a scene text recognition framework named CLIP-Llama, combining CLIP’s visual perception capabilities with Llama’s large language modeling abilities. CLIP-Llama includes two encoding–decoding branches: a visual branch and a cross-modal branch. The visual branch comprises a CLIP image encoder and a visual decoder. In contrast, the cross-modal branch includes a CLIP text encoder, a cross-modal decoder, and the Llama language model. The output from the visual branch undergoes further prediction through the cross-modal branch. The main contributions of this paper include (1) proposing a scene text recognition approach using the vision-language model CLIP and language model Llama, (2) introducing a threshold-based decision mechanism that enables score comparison and character-level masking, significantly reducing resource wastage, and (3) achieving state-of-the-art (SOTA) performance on mainstream benchmarks with the proposed CLIP-Llama.

## 2. Related Work

Scene Text Recognition (STR) has been a long-standing topic of interest and research [12]. With the widespread adoption of deep learning methods, their effectiveness in the field of STR has been widely validated. Based on the application of language awareness, we categorize STR methods into two types: language-agnostic methods and language-aware methods.

### 2.1. Language-Agnostic STR Methods

The mainstream approach to feature extraction in STR methods relies on CNN [13]. For instance, earlier STR methods [14] used VGG, while current STR methods employ ResNet [15] for improved performance. Various methods have been proposed to address STR based on the strength of CNN features. CTC-based methods [14] use Connectionist Temporal Classification (CTC) for sequence recognition. Segmentation-based methods approach STR as a semantic segmentation problem. Inspired by the success of Transformers [16] in natural language processing (NLP) tasks, the application of Transformers in STR has attracted increased attention. Vision Transformers (ViTs) [17] can directly process image patches without convolution, setting a precedent for using Transformer blocks to tackle computer vision problems and achieving prominent results. ViTSTR [18] attempts to utilize the feature representations from the ViT’s final layer for parallel character decoding. Additionally, some methods use Generative Adversarial Networks (GANs) [19] or segmentation networks to assist in text recognition. For example, Luo et al. [20] used GANs to mitigate background complexity in-text images, and Liu et al. [21] and Wang et al. [22] proposed multitask frameworks integrating text recognition and font mask generation using GANs. Generally, language-agnostic methods often struggle to recognize low-quality images due to a lack of language information.

### 2.2. Language-Aware STR Methods

Language information is beneficial for recognizing low-quality images. RNN-based methods [14] effectively capture dependencies between consecutive characters, which can be considered an implicit language model. However, they cannot perform decoding in parallel during training and inference. Recently, Transformer blocks have been introduced into CNN-based frameworks to facilitate language content learning. SRN [1] proposed a Global Semantic Reasoning Module (GSRM) to capture global semantic context through multiple parallel transmissions. ABINet [2] introduced a Bidirectional Cloze Network (BCN) [2] for explicit modeling of language information, further used for iterative correction. VisionLAN [23] proposed a visual reasoning module that captures visual and linguistic information by masking the input image at the feature level. NRTR [24] adopts a left-to-right autoregressive decoding approach, while PARSeq [25] uses different attention masks for more detailed semantic modeling.

### 2.3. Pre-Trained Models for STR

To enhance the performance of STR methods, several pre-trained STR research efforts have been proposed [26,27]. They generally fall into two categories: encoder pre-training and entire model pre-training. Encoder pre-training uses a large amount of unlabeled real images to guide the encoder in learning real image representations, often through self-supervised learning methods such as Masked Autoencoders (MAE) [28] or contrastive learning. The trained encoder can then be applied more effectively to different downstream tasks. For example, SeqCLR [29] introduced a sequence-to-sequence contrastive learning framework for text images, and CCD [27] incorporated glyph pseudo-labels to guide the encoder’s focus on character foregrounds. MAERec [28] used a ViT-based STR model, demonstrating that the model can utilize unlabeled images through masked image modeling tasks. By contrast, entire model pre-training typically involves pre-training part or all of the model and then fine-tuning it as a whole. For instance, TrOCR [30] learned visual representations by pre-training on printed text images and fine-tuning on synthetic scene text images. Additionally, it incorporates BERT-style pre-training. MaskOCR [31] follows a three-stage approach, including encoder pre-training, decoder pre-training, and full-model fine-tuning. Recent research also evaluates pre-training on synthetic data and fine-tuning on real data. These methods primarily pre-train on synthetic text images, but the domain gap between synthetic and real text images remains a significant factor limiting their real-world performance. Given the CLIP encoder’s ability to better extract real image information and enhance language information via the Llama language model, we designed the CLIP-Llama network to perform STR tasks based on these advantages.

## 3. Method

**Figure 1:**
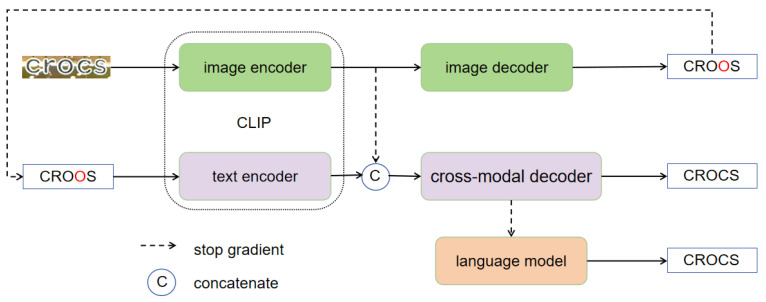
The overall framework of CLIP-Llama. It comprises a visual branch and a cross-modal branch. The cross-modal branch refines and corrects the predictions from the visual branch to produce the final output.

CLIP-Llama consists of two encoding–decoding branches: a visual branch and a cross-modal branch. The visual branch includes the CLIP image encoder and a visual decoder, while the cross-modal branch consists of the CLIP [3] text encoder, a cross-modal decoder, and the Llama language model [10]. The output from the visual branch undergoes further prediction through the cross-modal branch. Specifically, as shown in Figure 1, we utilize the CLIP visual encoder and text encoder to encode the image and text, with the output being decoded by the visual decoder and the cross-modal decoder. For predictions from the cross-modal decoder, we set a confidence threshold: predictions that exceed the threshold are directly output, while those below the threshold are re-decoded using Llama to produce the final output.

### 3.1. Image Encoder and Text Encoder of CLIP

**Figure 2:**
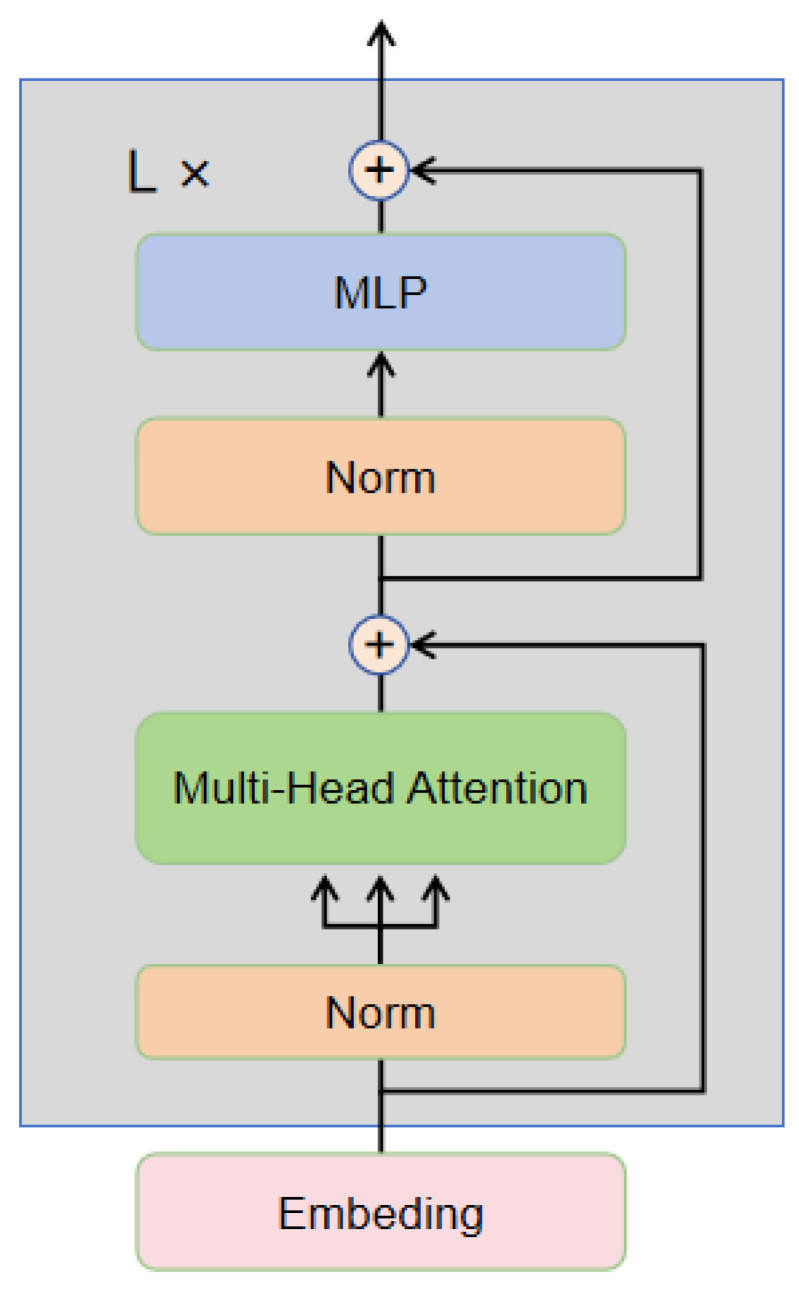
Encoder framework.

The image encoder in CLIP primarily uses a Vision Transformer (ViT) [17], specifically a 24-layer Transformer Encoder [16] structure. The internal structure of this encoder operates shown in Figure 2.

The core idea behind ViT is to divide an image into smaller patches and then process these patches as a sequence input to a Transformer model. This process can be divided into the following steps: Divide the input image into embedded patches. The input image is divided into *N* patches, and each patch is mapped to a fixed-dimensional embedding through a linear projection. Let each embedding be represented as $x_{i}$.

(1) $$z_{0}^{i}=x_{p}^{i}E,\;x_{p}^{i}\in R^{N},\;E\in R^{N\times D}$$

In this context, $x_{p}^{i}$ is the vector of the *i*-th image patch, and *E* is the learnable linear projection matrix. Normalize it through a normalization layer to obtain the initial input $h_{0}$.

(2) $$h_{0}=\mathrm{Norm}\left(x\right)\;h_{0}\in R^{N\times D}$$

Multi-Head Self-Attention The first part of the encoder consists of a multi-head self-attention layer. Let $h_{l}$ be the input features for layer L, and the multi-head attention computation process is as follows:

Calculate the query Q, key K, and value V: $Q=h_{0}W_{Q},K=h_{0}W_{K},V=h_{0}W_{V}$

(3) $$\mathrm{Attention}\left(Q,K,V\right)=\mathrm{softmax}\left(\frac{QK^{T}}{\sqrt{d_{k}}}\right)V$$

Add the output of multi-head attention to the input through a residual connection.

(4) $$h_{l+1}=h_{l}+\mathrm{Attention}\left(Q,K,V\right)$$

The output of multi-head attention is processed through a normalization layer to obtain the new feature $h_{l+1}^{\prime }$:

(5) $$h_{l+1}^{\prime }=\mathrm{Linear}(\mathrm{Norm}(h_{l+1}))\;+\;h_{l+1}$$

The above steps are repeated L times in the Transformer Encoder. After stacking L layers, the encoder finally outputs the feature representation $h_{L}$.

The text encoder is similar to the image encoder, except that the input is text instead of images. We denote the output of the text encoder as $g_{L}$. By concatenating the outputs of the image encoder and the text encoder, we create the input for the multimodal decoder.

(6) $$F_{i}=h_{L}\in R^{L_{i}\times D},F_{t}=g_{L}\in R^{L_{t}\times D},F_{c}={\left[\begin{array}{cc} F_{i}^{T} & F_{t}^{T} \end{array}\right]}^{T}\in R^{L_{c}\times D}$$

$F_{i}$ serves as the input for the visual decoder, and $F_{c}$ serves as the input for the multimodal decoder. $L_{t}$ represents the length of the text sequence, $L_{i}$ is the length of the image token sequence, D denotes the dimensionality of the joint image-text embedding space, and the cross-modal sequence length $L_{c}=L_{i}+L_{t}$.

### 3.2. Image Decoder and Cross-Modal Decoder

**Figure 3:**
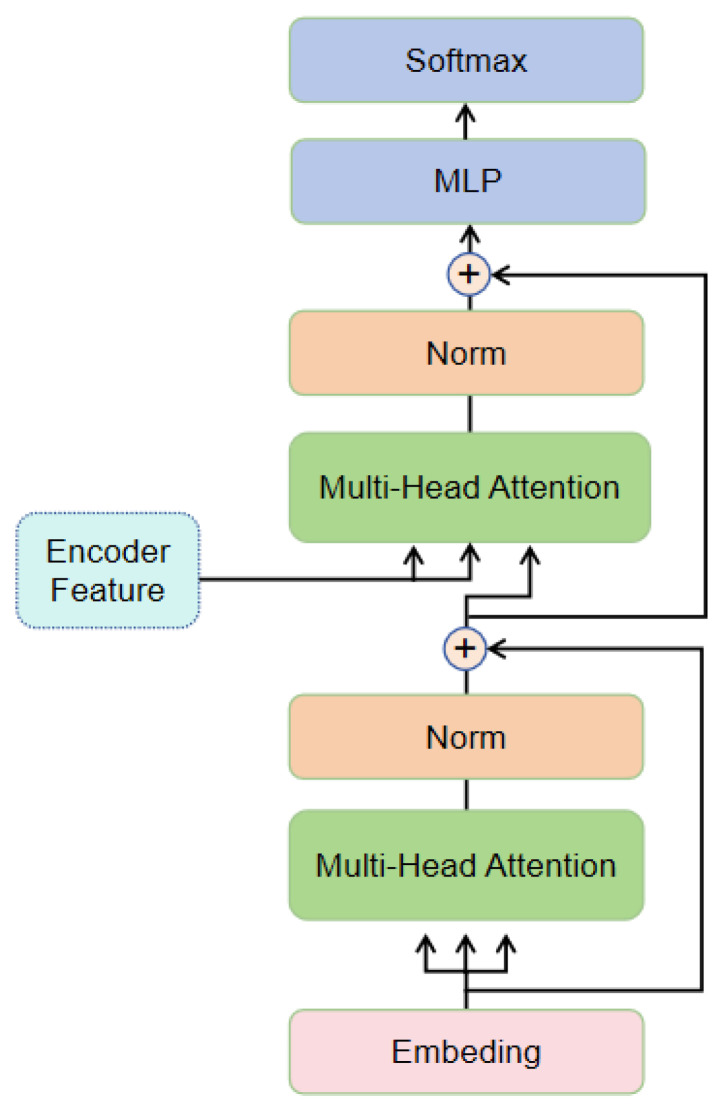
Decoder framework.

The image decoder and cross-modal decoder use the same structure. The difference is that the visual decoder receives features from the visual encoder, while the cross-modal decoder receives concatenated features from both the visual encoder and the text encoder. Its structure is shown in Figure 3.

It adopts a transformer decoder design along with the Permutation Sequence Modeling (PSM) technique, allowing predicted characters to have arbitrary dependencies on input context during training. The visual decoder and cross-modal decoder have the same architecture but different inputs. For the visual decoder, c is not required. They receive the following inputs: learnable positional queries *p*, input context *c*, and randomly generated attention mask M. The decoder outputs predictions y. The decoding stage can be represented as

(7) y=DEC(p,c,M,F)

The first Multi-Head Attention (MHA) in Figure 2 performs context-position attention:

(8) $$m_{1}=\mathrm{softmax}\left(\frac{pc^{T}}{\sqrt{D}}+M\right)c+p.$$

The second MHA attends to feature-position attention:

(9) $$m_{2}=\mathrm{softmax}\left(\frac{m_{1}F^{T}}{\sqrt{D}}\right)F+m_{1}$$

For simplicity, we omitted the input and output linear transformations as well as the normalization operations in the attention mechanism of Equations (8) and (9). Then, we use $m_{2}$ for the next prediction *y*, recording the prediction score for each character.

(10) $$y=\mathrm{Linear}(\mathrm{MLP}\left(m_{2}\right)+m_{2})$$

The prediction y and the prediction scores will be sent to the threshold judgment and the language model.

### 3.3. Threshold Judgement and Language Model

**Figure 4:**
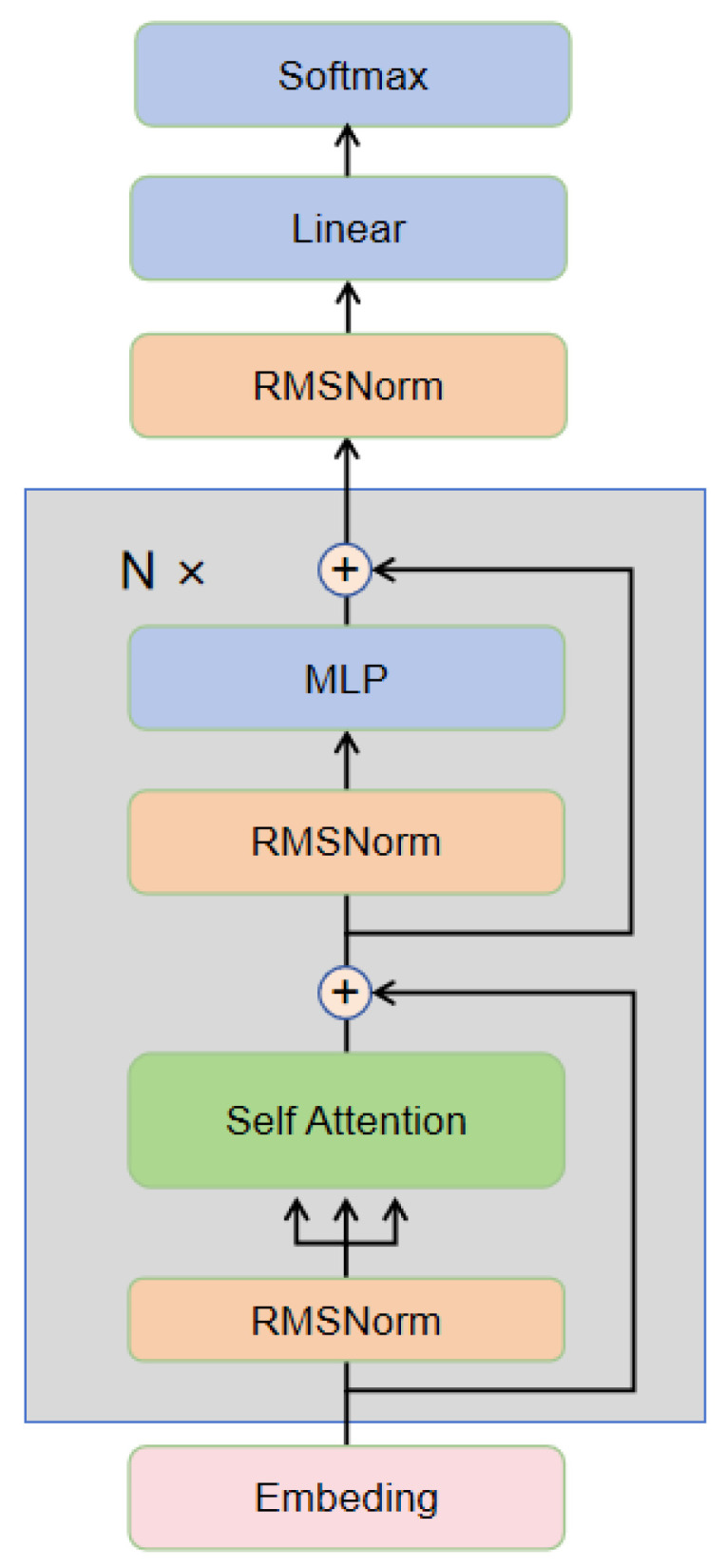
Decoder framework.

In general, with the support of the CLIP visual and text encoders, the multimodal decoder achieves relatively high accuracy. If each prediction is passed through the language model for correction, it will lead to resource wastage. Therefore, we implement a threshold judgment mechanism. If all characters in the multimodal decoder’s prediction have confidence scores exceeding a preset threshold, the prediction from the multimodal decoder is directly output. If any character’s confidence score falls below the threshold, that character is masked, and the masked result is sent to the Llama language model for re-decoding. The final output is then generated based on Llama’s prediction. For the language model, we selected LLaMA2 [11], a large language model developed by Meta AI based on the Transformer architecture [16], primarily utilizing the Transformer decoder. The structure of this framework is illustrated as shown in Figure 4.

The input passes through the embedding layer to obtain the embedded representation. The input embeddings are normalized through the RMSNorm layer. The self-attention layer uses a Grouped Multi-Query Attention mechanism with Q, K, and V caching.

(11) $$h_{0}=\mathrm{Embedding}(\mathrm{Input})$$

Calculate the query Q, key K, and value V: $Q=h_{0}W_{Q},K=h_{0}W_{K},V=h_{0}W_{V}$

(12) $$\mathrm{Attention}\left(Q,K,V\right)=\mathrm{softmax}\left(\frac{QK^{T}}{\sqrt{d_{k}}}\right)V$$

Add the output of multi-head attention to the input through a residual connection.

(13) $$h_{1}=h_{0}+\mathrm{Attention}\left(Q,K,V\right)$$

The output of the attention layer is normalized again through the RMSNorm layer.

(14) $$h_{2}=\mathrm{RMSNorm}(h_{1})$$

The feedforward network uses the SwiGLU activation function, and the output of the feedforward network is connected to the input through a residual connection.

(15) $$h_{3}=\mathrm{Linear}\left(\mathrm{MLP}\left(h_{2}\right)+h_{1}\right)$$

After repeating the above steps N times, the final output passes through RMSNorm, a linear transformation, and Softmax to obtain the final output prediction.

(16) $$y=\mathrm{Softmax}(\mathrm{Linear}\left(\mathrm{RMSNorm}\left(h_{3}\right)\right))$$

The LLaMA model structure follows the typical Transformer encoder architecture, incorporating mechanisms such as Grouped Multi-Query Attention, SwiGLU feedforward network, and RMSNorm. It achieves model expressiveness through multiple stacked layers.

### 3.4. Supervised Training Loss

The loss is calculated as

CLIP-Llama is optimized to minimize the sum of cross-entropy losses (CE(·)) for the visual branch and the cross-modal branch, Cross-Entropy Loss is a loss function used to evaluate the output of classification models, especially suitable for multi-class problems. It measures the model’s performance by calculating the difference between predicted and true distributions. The smaller the cross-entropy loss, the closer the model’s predictions are to the true labels. For multi-class classification, the cross-entropy loss extends to

(17) $$\mathrm{CE}\left(\right)=-\frac{1}{N}\sum_{i=1}^{N}\sum_{j=1}^{C}y_{ij}\log\left(p_{ij}\right)$$

where *C* is the number of classes, $y_{ij}$ is the true label indicating whether the *i*-th sample belongs to class *j*, and $p_{ij}$ is the predicted probability for class *j* for that sample.

The loss of CLIP-Llama is calculated as

(18) $$L=\mathrm{CE}\left(y^{i},\hat{y}\right)+\mathrm{CE}\left(y^{c},\hat{y}\right)$$

where $\hat{y}$ represents the ground truth, $y^{i}$ is the prediction of the visual decoder, and $y^{c}$ is the prediction of the cross-modal decoder.

In summary, CLIP-Llama consists of a visual branch and a cross-modal branch. To fully utilize the functionality of both branches, we employ a dual-prediction and optimized decoding scheme during inference. The visual branch first performs autoregressive decoding, where each subsequent output depends on the previous predictions. Then, the cross-modal branch addresses potential discrepancies between the visual predictions and text semantics. The decoder’s output serves as an intermediate prediction. For results with confidence scores below the threshold, the Llama language model is used for fill-in-the-blank prediction to enhance recognition accuracy.

## 4. Experiment

### 4.1. Dataset

**Figure 5:**
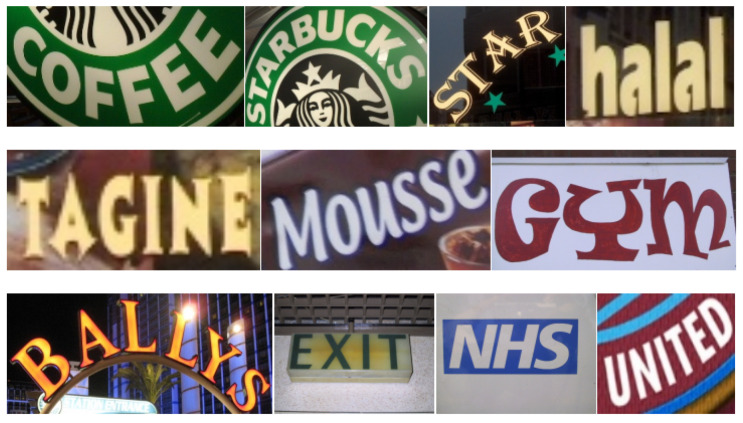
Part of the dataset images.

Previous studies on training datasets have shown that real training data can lead to better performance compared to commonly used synthetic data such as MJSynth (MJ, 9M samples) [32] and SynthText (ST, 6.9M samples) [33]. Therefore, we primarily utilize real data for training. Some examples can be seen in Figure 5. Specifically, we use COCO-Text (COCO) [34], RCTW17 [35], Uber-Text (Uber) [36], ArT [37], LSVT [38], MLT19 [39], ReCTS [40], TextOCR [41], and Open Images annotations [42] from the OpenVINO toolkit [43]. These real datasets collectively comprise 3.3 million images. Evaluation benchmarks include IIIT5K [44], CUTE80 [45], Street View Text (SVT) [46], SVT-Perspective (SVTP) [47], ICDAR 2013 (IC13) [48], ICDAR 2015 (IC15) [49], as well as two proprietary datasets—HOST and WOST [23]. Additionally, we utilize three recent large-scale benchmarks: COCO-Text (9.8K samples; low-resolution, occluded text) [34], ArT (35.1K samples; curved and rotated text) [37], and Uber-Text (80.6K samples; vertical and rotated text) [36].

### 4.2. Experimental Configuration

Label preprocessing follows the method used in previous work. During training, we set the maximum label length to T = 26 and used a character set size of S = 94, which includes a mix of uppercase and lowercase alphanumeric characters and punctuation. Image preprocessing is conducted: images are first augmented, resized, and finally normalized to the range [−1, 1]. The augmentation set primarily includes RandAugment operations, excluding sharpness adjustments. All images are resized unconditionally to 224 × 224 pixels. During inference, only lowercase letters and numeric characters are used, resulting in C = 36. The evaluation metric is word accuracy, where a prediction is considered correct only if all characters match strictly at every position. Model parameters: images are divided into 224 × 224 pixels, segmented into 256 small patches of 14 × 14 pixels each, with an embedding dimension of 512. The encoder has 12 heads, a depth of 24, and a width of 768, while the decoder has 8 heads, a depth of 1, and a width of 768. Other parameters are consistent with CLIP4STR. The Llama model parameters remain unchanged. Learning Strategy: We apply warm-up and cosine learning rate decay strategies. The learning rate for the CLIP encoder is set to $8.4\times 10^{-5}\times \frac{batch\;size}{512}$. For models trained from scratch (decoder), the learning rate is multiplied by 19.0, with a batch size 960. For real data, we train for 10 epochs. For synthetic data, we train for 5 epochs. We use the AdamW [50] optimizer with a weight decay value of 0.2. All experiments are conducted with mixed precision training on 8 NVIDIA GeForce RTX 4090 GPUs.

### 4.3. Comparison Experiment

Comparison with State-of-the-Art Techniques: We compared CLIP-Llama and previous state-of-the-art (SOTA) methods on 8 common STR benchmarks as shown in Table 1. CLIP-Llama significantly outperforms previous methods, achieving new SOTA performance. It is noteworthy that CLIP-Llama performs exceptionally well on irregular text datasets such as IC13, IC15 (incidental scene text), SVTP (perspective scene text), HOST (severely occluded scene text), and WOST (weakly occluded scene text). This is because CLIP demonstrates robust recognition of both regular and irregular text. CLIP-Llama exhibits excellent reading ability on occluded datasets, outperforming previous SOTA by 2.53% and 1.32% on HOST and WOST, respectively. This capability can be attributed to the pre-trained language model Llama, which utilizes textual semantics to infer erroneous or missing characters.

**Figure 6:**
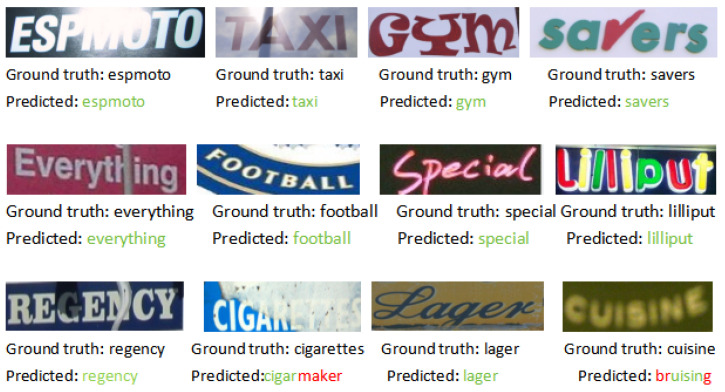
The model’s text recognition results.

In addition to small-scale general benchmarks, we evaluated CLIP-Llama on three larger, more challenging benchmarks. These benchmarks primarily consist of the irregular text of various shapes, low-resolution images, rotations, etc. The results are shown in Table 2, where we highlight the best results in bold, further demonstrating the strong generalization ability of CLIP-Llama. It significantly outperforms previous SOTA methods, with a notable improvement of 1.19% accuracy on the COCO dataset and a 0.95% accuracy improvement on the ArT dataset compared to the previous SOTA. Figure 6 shows the qualitative results of CLIP-Llama compared to CLIP4STR on the test set, demonstrating relative improvement over the original CLIP4STR model. Due to various degrees of blurriness, occlusion, and lighting issues, a purely visual model could not recognize certain images. However, thanks to CLIP’s powerful feature extraction capabilities for real images and Llama’s strong semantic correction abilities, CLIP-Llama significantly enhances accuracy in STR tasks, thereby validating its generalization capabilities.

Before using the language model Llama, we scored the predicted characters based on their confidence levels. When the score was below a certain threshold, the characters were masked. We conducted experiments with different confidence thresholds, specifically 0.7, 0.8, and 0.9, as shown in Table 3. The best results are highlighted in bold. Through experimentation, we found that setting the threshold to 0.8 yielded the best overall performance.

While our model has achieved excellent performance, there are still some inaccuracies in the recognition results. This may be due to the ambiguity in the language model or because the images are pretty blurry, leading to the visual model being unable to identify the results accurately. Overall, CLIP-Llama fine-tunes the pre-trained CLIP and Llama models and effectively transfers the knowledge of CLIP and Llama to the STR task. These results support our motivation that CLIP-Llama has strong scene text perception capabilities and can correct misrecognition, serving as an effective scene text reader.

## 5. Conclusions

In conclusion, we propose a novel text recognition method called CLIP-Llama, which leverages CLIP and Llama for STR. It features a dual encoder–decoder architecture: a visual branch for initial prediction, a cross-modal branch for refinement, and a language model correction module. In this approach, we first use the pre-trained visual language model CLIP to extract image features and make initial predictions. Then, the uncertain predictions are refined using the powerful language model Llama, pre-trained on a large corpus, to generate high-confidence predictions. Through this process, CLIP-Llama achieves state-of-the-art results on 11 STR benchmarks, demonstrating its effectiveness as a robust scene text reader and the benefit of visual language pre-training for STR. We envision CLIP-Llama as a simple yet powerful baseline for future STR research.

## Figures and Tables

**Table 1:** Word Accuracy on 8 Common Benchmarks. Bold highlights the best results. Benchmark datasets (B)—SVT, IIIT5K, SVTP, CUTE80, IC13, and IC15. MJ+ST represents training with synthetic datasets, B represents benchmark datasets, Real represents real datasets, and Union14M-L [51] represents large-scale datasets. In the “type” column, “V” represents using only the visual model, and “VL” represents using both the visual and language models.

Method	Type	Train Data	III5k 3000	SVT 647	IC13 1015	IC15 1811	IC15 2077	SVTP 645	CUTE 288	HOST 2416	WOST 2416
PlugNet	V	MJ+ST	94.4	92.3	95.0	-	82.2	84.3	85.0	-	-
ASTER	V	MJ+ST	93.4	89.5	-	76.1	-	78.5	79.5	-	-
SRN	VL	MJ+ST	94.8	91.5	-	82.7	-	85.1	87.8	-	-
TextScanner	V	MJ+ST	95.7	92.7	94.9	-	83.5	84.8	91.6	-	-
SE-ASTER	V	MJ+ST	93.8	89.6	92.8	80.0	-	81.4	83.6	-	-
RCEED	VL	MJ+ST+B	94.9	91.8	-	-	82.2	83.6	91.7	-	-
TRBA	V	MJ+ST	92.1	88.9	-	86.0	-	89.3	89.2	-	-
	VisionLAN	VL	MJ+ST	95.8	91.7	-	83.7	-	86.0	88.5	50.3 70.3
ABINet	VL	MJ+ST	96.2	93.5	-	86.0	-	89.3	89.2	-	-
ViTSTR-B	V	MJ+ST	88.4	87.7	92.4	78.5	72.6	81.8	81.3	-	-
LevOCR	VL	MJ+ST	96.6	92.9	-	86.4	-	88.1	91.7	-	-
MATRN	VL	MJ+ST	96.6	95.0	95.8	86.6	82.8	90.6	93.5	-	-
PETR	V	MJ+ST	95.8	92.4	97.0	83.3	-	86.2	89.9	-	-
DiG-ViT-B	VL	MJ+ST	96.7	94.6	96.9	87.1	-	91.0	91.3	74.9	82.3
TrOCR	VL	MJ+ST+B	94.1	96.1	97.3	88.1	84.1	93.0	95.1	-	-
SIGA	VL	MJ+ST	96.6	95.1	96.8	86.6	83.0	90.5	93.1	-	-
PARSeq	VL	MJ+ST	97.0	93.6	96.2	86.5	82.9	88.9	92.2	-	-
CLIP4STR-L	VL	MJ+ST	98.0	95.2	96.9	87.7	84.5	93.3	95.1	82.7	88.8
MAERec-B	VL	Union14M-L	98.5	97.8	98.1	-	89.5	94.4	98.6	-	-
IGTR-PR	VL	MJ+ST	97.6	95.2	97.6	88.4	88.4	91.6	95.5	-	-
MGP-STR(Fuse)	VL	MJ+ST	96.4	94.7	97.3	87.2	87.2	91.0	90.2	-	-
CAM-Base	VL	MJ+ST	97.4	96.1	97.2	87.8	87.8	90.6	92.4	-	-
SVIPTRv2-B	VL	MJ+ST	94.8	94.2	97.0	88.0	88.0	90.0	90.2	-	-
DiG-ViT-B	VL	Real	97.6	96.5	97.6	88.9	-	92.9	96.5	62.8	79.7
ViTSTR-S	V	Real	97.9	96.0	97.8	89.0	87.5	91.5	96.2	64.5	77.9
ABINet	VL	Real	98.6	98.2	98.0	90.5	88.7	94.1	97.2	72.2	85.0
PARSeq	VL	Real	99.1	97.9	98.4	90.7	89.6	95.7	98.3	74.4	85.4
NRTR+DPTR	VL	Real	99.2	97.8	98.1	91.8	90.6	95.7	98.6	-	-
CLIP4STR-L	VL	Real	**99.5**	**98.5**	98.5	91.3	90.8	97.4	**99.0**	79.8	89.2
CLIP-Llama(Ous)	VL	Real	99.47	98.45	**98.52**	**91.99**	**91.43**	**97.67**	98.96	**82.33**	**90.52**

**Table 2:** Word accuracy on three large-scale benchmarks.

Method	Train Data	COCO 9825	ArT 35,149	Uber 80,551
ViTSTR-S	MJ+ST	56.4	66.1	37.6
TRBA	MJ+ST	61.4	68.2	38.0
ABINet	MJ+ST	57.1	65.4	34.9
PARSeq	MJ+ST	64.0	70.7	42.0
MPSTR	MJ+ST	64.5	69.9	42.8
CLIP4STR-L	MJ+ST	67.0	73.7	44.5
DiG-ViT-B	Real	75.8	-	-
ViTSTR-S	Real	73.6	81.0	78.2
TRBA	Real	77.5	82.5	81.2
ABINet	Real	76.5	81.2	71.2
PARSeq	Real	79.8	84.5	84.1
MPSTR	Real	80.3	84.4	84.9
CLIP4STR-L	Real	81.9	85.9	87.6
CLIP-Llama(Ours)	Real	**83.09**	**86.85**	**87.67**

**Table 3:** Experimentation with different confidence thresholds.

Threshold	Train Data	III5k 3000	SVT 647	IC13 1015	IC15 1811	IC15 2077	SVTP 645	CUTE 288
0.7	Real	99.31	98.12	98.10	91.12	91.34	**97.69**	98.37
0.8	Real	**99.47**	**98.45**	**98.52**	**91.99**	91.43	97.67	**98.96**
0.9	Real	99.35	98.20	98.27	91.67	**91.54**	97.54	98.76

## Data Availability

Data are contained within the article.
